# The role of ubiquitination and deubiquitination in tumor invasion and metastasis

**DOI:** 10.7150/ijbs.69411

**Published:** 2022-03-06

**Authors:** Shuangze Han, Ruike Wang, Yangnan Zhang, Xiaoying Li, Yu Gan, Feng Gao, Pengfei Rong, Wei Wang, Wei Li

**Affiliations:** 1 Department of Radiology, The Third Xiangya Hospital of Central South University, Changsha, Hunan 410013, China; 2 Cell Transplantation and Gene Therapy Institute, The Third Xiangya Hospital, Central South University, Changsha, Hunan, 410013, China; 3 Department of Ultrasonography, The Third Xiangya Hospital of Central South University, Changsha, Hunan, 410013, China

**Keywords:** ubiquitination, E3 ubiquitin ligase, deubiquitinase, metastasis

## Abstract

Ubiquitination is vital for multiple cellular processes via dynamic modulation of proteins related to cell growth, proliferation, and survival. Of the ubiquitination system components, E3 ubiquitin ligases and deubiquitinases have the most prominent roles in modulating tumor metastasis. This review will briefly summarize the observations and underlying mechanisms of multiple E3 ubiquitin ligases and deubiquitinases to regulate tumor metastasis. Further, we will discuss the relationship and importance between ubiquitination components and tumor progression.

## A brief overview of the ubiquitination process

**Figure 1:**
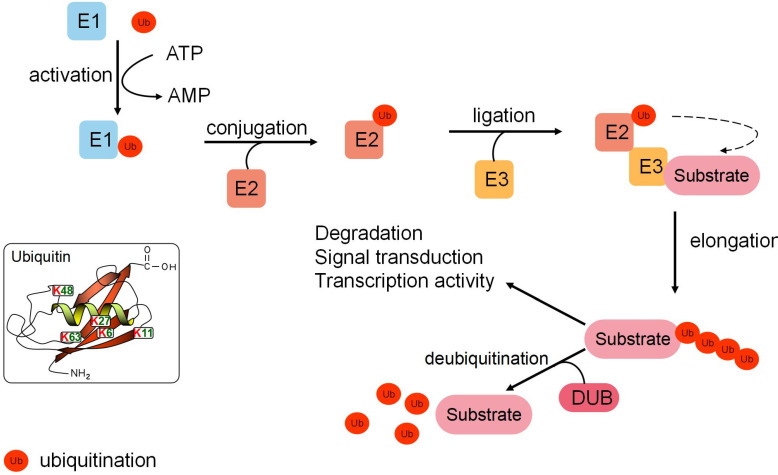
** A brief overview of the ubiquitination pathway.** Ubiquitination is catalyzed by a three-enzyme cascade composed of the E1 Ub-activating enzyme, the E2 Ub-conjugating enzyme, and the E3 Ub ligase. The E3 ligase selectively recognizes substrate proteins by forming an iso-peptide bond and recruits the Ub-E2 complex to catalyze the transfer of Ub to the substrate from E2. Elongation and distinct polyubiquitin chains are involved in protein degradation, signal transduction, and transcriptional activity. Deubiquitinases remove Ub moieties from substrate proteins with high specificity and reverse Ub signals to maintain cellular dynamic ubiquitination.

Protein ubiquitination is a dynamic, multifaceted post-translational modification involved in multiple cellular processes. Ubiquitin (Ub), a 76-amino acid protein, features seven lysine residues (K6, K11, K27, K29, K33, K48, and K63), which can each be ubiquitinated to form distinctive forms of polyubiquitin chains. Different polyubiquitin chains mediate distinct signaling pathways to determine the fate of substrate proteins 1. For example, K48/K11-linked chains are responsible for targeting substrate proteins for proteasomal degradation, while other chains perform non-degradative roles in controlling protein interactions, cellular localization, and signaling transduction (Fig. 1).

Ubiquitination is catalyzed by a three-enzyme cascade composed of the E1 Ub-activating enzyme, the E2 Ub-conjugating enzyme, and the E3 Ub ligase. E1 recruits and activates Ub by utilizing the energy of ATP. Activated Ub is transferred to E2, which can transfer Ub to the target substrate. E3 Ub ligase selectively recognizes a substrate protein by forming an iso-peptide bond between the COOH-terminal glycine of Ub and a lysine residue of the substrate. In addition, E3 Ub ligase recruits the E2-Ub complex and catalyzes the transfer of Ub to the substrate from E2 2. Different subtypes of E3s (the RING type E3s, the HECT type E3s, and the RBR type E3s) are the most critical component of the ubiquitination cascade for the substrate recognition capacity 2. Meanwhile, ubiquitination is a dynamic and reversible process. Deubiquitinases (DUBs) can act as an “eraser” that reverses Ub signals. Most DUBs remove Ub moieties from proteins to prevent substrate proteins from degradation. However, some proteasome-related DUBs, including USP14, UCHL5, PSMD14, and PSMD7, are localized in 19S particles of the proteasome 3. The roles of these DUBs are to deubiquitinate the substrates and facilitate their degradation in 20S particles of the proteasome. Alternatively, DUBs can alter signals by non-degradation ubiquitination 4 (Fig. 1).

The Ub cascade and DUBs synergistically regulate protein turnover and function in numerous signaling pathways to maintain cellular invasion and metastasis.

## A novel insight into tumor metastasis

Tumor metastasis remains the primary cause of cancer-associated mortality. Metastasis involves tumor cell motility, intravasation into the adjacent tissues, circulation, and extravasation to distant organs. Simultaneously, the process is caused by genome instability where cancer cells can reprogram tumor metabolism, resist cell death, avoid immune destruction, and constitute the tumor microenvironment 5, 6. The epithelial-mesenchymal transition (EMT) is an equally crucial determinant during the metastatic cascade 7.

Ubiquitination and deubiquitination broadly participate in various processes involved in protein modification and regulation. Aberrant dysregulation induces tumorigenesis. This review is primarily focused on recent novel observations and underlying mechanisms concerning E3 ligases and DUBs in order to contribute to further elucidating the role of ubiquitination and deubiquitination in tumor invasion and metastasis.

## E3 Ub ligases inhibiting metastasis

### F-box and WD-repeat domain-containing 2 (FBXW2)

**Figure 2:**
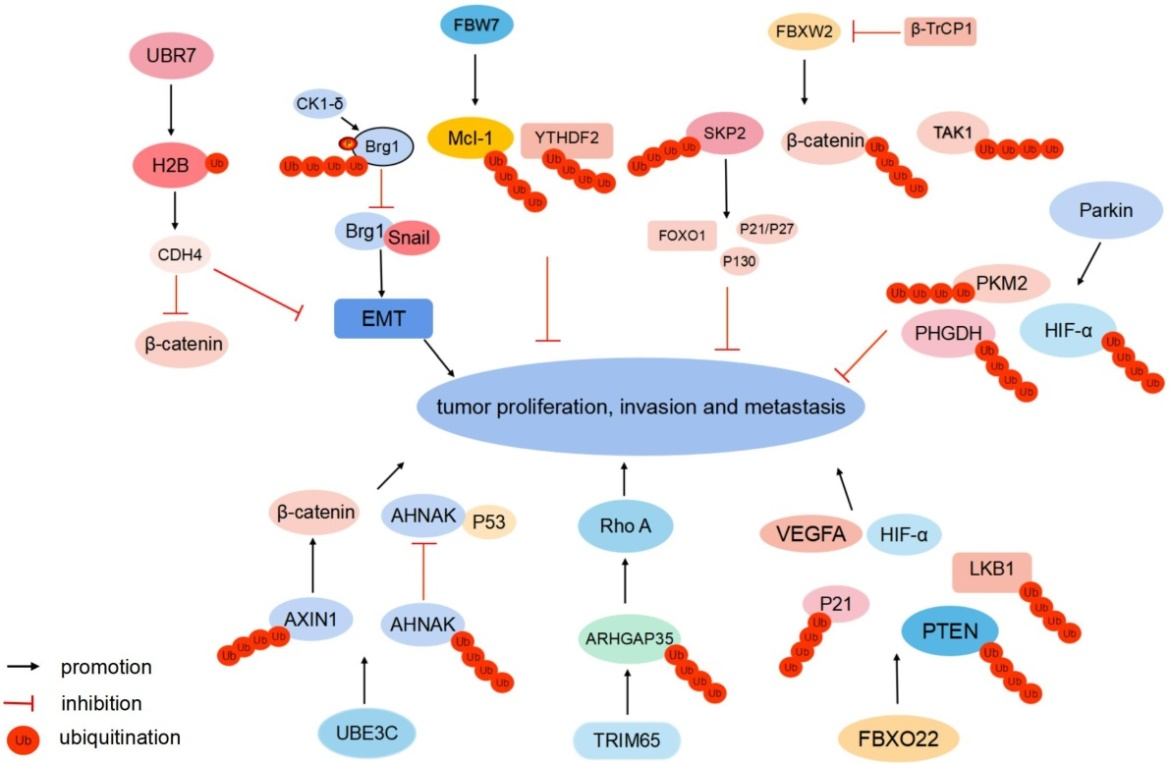
** Different E3 Ub ligases regulate tumor metastasis.** FBXW2 targets SKP2, β-catenin, and TAK1, FBW7 targets Brg1, Mcl-1, and YTHDF2, and Parkin targets HIF-1α, PHGDH, and PKM2 for ubiquitination degradation to suppress tumor proliferation and metastasis, respectively. UBR7 monoubiquitinates histone H2B to suppress EMT and nuclear β-catenin. UBE3C targets AHNAK and AXIN1 for ubiquitination-induced degradation. TRIM65 ubiquitinates ARHGAP35, FBXO22 ubiquitinates nuclear PTEN and p21 to enhance cancer cell migration, respectively. FBXO22 mediates Lys-63-linked LKB1 ubiquitination. FBXO22 upregulates HIF-1α and VEGFA to promote tumor proliferation and metastasis.

FBXW2 is a substrate recognition receptor in the SKP1-Cullin1-F-box protein (SCF) E3 Ub ligase complex. FBXW2 suppresses proliferation and invasion of lung cancer cells by targeting S phase kinase-associated protein 2 (SKP2) and β-catenin (Fig. 2). FBXW2 is downregulated and negatively correlated with β-catenin in lymph-node metastasis 8, 9. Meanwhile, FBXW2 is a novel substrate of β-transducin repeat-containing protein 1 (β-TrCP1). Following growth factor stimulation, β-TrCP1 targets FBXW2 for ubiquitination degradation. The accumulation of SKP2 subsequently leads to the degradation of tumor suppressors and apoptosis-inducing substrates, such as p21, p27, p130, and FOXO1, to promote tumor cell proliferation, growth, and survival 8. The β-TrCP-FBXW2-SKP2 signaling cascade forms the oncogene (β-TrCP1)-tumor suppressor gene (FBXW2)-oncogene (SKP2) axis that regulates the growth and survival of lung cancer cells via targeting each other for degradation, which is a process of crucial crosstalk among F-box proteins. In hepatocellular carcinoma (HCC), FBXW2 targets transforming growth factor-β-activated kinase 1 (TAK1) for K48-linked polyubiquitination and degradation to inhibit cancer progression 10 (Fig. 2). Clinically, FBXW2 levels could be a significant indicator of the prognosis and survival of patients with cancer.

### F-box and WD repeat domain-containing 7 (FBW7)

FBW7 is a substrate recognition receptor of the SCFFBW7 E3 Ub ligase complex. It functions as a tumor suppressor and mediates ubiquitination-induced degradation of various oncogenic proteins, including c-MYC, NOTCH, c-JUN, and cyclin E 11. In gastric cancer, transcription activator Brg1 (Brg1) binds to the promoter of Snail, which subsequently promotes EMT and metastasis. Meanwhile, Brg1 is a Ub substrate of the SCFFBW7 E3 ligase complex. Casein kinase 1 (CK1)δ-mediated phosphorylation at Ser31/Ser35 sites of Brg1 strengthens FBW7-binding capacity, thus accelerating the ubiquitination of Brg1^12^ (Fig. 2). Methylation induces epigenetic silencing of the FBW7 gene. Decitabine (DAC) epigenetically activates FBW7 expression via its demethylation. DAC-activated FBW7 promotes myeloid leukemia cell differentiation protein Mcl-1 ubiquitination and degradation to suppress lung cancer growth 13. YTH domain-containing family protein 2 (YTHDF2) is the N6-methyladenosine (m6A) reader protein and promotes the decay of the m6A-modified mRNAs. FBW7 can degrade YTHDF2 by ubiquitination and suppress the propagation of ovarian cancer 14 (Fig. 2).

### Putative E3 Ub-protein ligase UBR7 (UBR7)

UBR7 belongs to the Ub-protein ligase E3 component N-recognin (UBR) family and has a unique plant homeodomain (PHD) finger 15. PHD fingers are central “readers” of histone post-translational modifications 16. UBR7-PHD finger monoubiquitinates histone H2B in triple-negative breast tumors at lysine 120 (H2BK120Ub). H2BK120Ub enhances cadherin-4 (CDH4) transcription activity and expression level 15. CDH4 overexpression notably suppresses EMT and reduces cellular proliferation, migration, and invasion 15 (Fig. 2). Meanwhile, CDH4 can regulate the Wnt/β-catenin signaling pathway. It alters the nuclear localization of β-catenin to the cytoplasm, which downregulates β-catenin target genes, including AXIN2, G1/S-specific cyclin-D1, C-MYC, COX2, and MMP7, to suppress tumor metastasis 17, 18.

### Parkin

Notably, as an E3 Ub ligase, Parkin can degrade substrate proteins associated with Parkinson's Disease (PD) 19. Meanwhile, Parkin acts as a tumor suppressor, and its expression is downregulated in various tumors 20. Parkin is an E3 Ub ligase for hypoxia-inducible factor 1α (HIF-1α) and can ubiquitinate HIF-1α at lysine 477 (K477), inhibit HIF-1α transcriptional activity, and induce its degradation to suppress breast cancer cells invasion and metastasis 21, 22. Parkin regulates HIF-1α in a Von Hippel-Lindau-independent manner, unveiling an additional layer of regulation for HIF-1α in cells. Phosphoglycerate dehydrogenase (PHGDH) is the first rate-limiting enzyme of serine synthesis. PHGDH overexpression activates serine synthesis to promote cancer progression. Parkin expression is inversely correlated with PHGDH expression in breast and lung cancer. Parkin interacts with PHGDH and ubiquitinates PHGDH at lysine 330, leading to PHGDH degradation to suppress serine synthesis 23. In intrahepatic cholangiocarcinoma (ICC), Parkin targets pyruvate kinase PKM2 for ubiquitination degradation to suppress migration and proliferation 24 (Fig. 2).

## E3 Ub ligases promoting metastasis

### Ub-protein ligase E3C (UBE3C)

UBE3C belongs to the HECT family of E3 Ub ligases. It functions as a tumor promoter and is aberrantly expressed in breast cancer 25, hepatocellular carcinoma 26, and renal cell carcinoma 27. In non-small cell lung cancer (NSCLC) tissues, UBE3C maintains cancer stemness by ubiquitinating and promoting neuroblast differentiation-associated protein AHNAK (AHNAK) degradation 28. AHNAK is a cofactor that assists P53 binding to stemness-related gene promoters. UBE3C-mediated degradation of AHNAK abrogates P53-AHNAK complex-mediated inhibition of gene expression, which enhances lung cancer cell stemness and NSCLC growth and metastasis 28, 29. UBE3C targets AXIN1 for ubiquitination degradation to activate β-catenin signaling in gastric cancer 30 (Fig. 2).

### Tripartite motif-containing protein 65 (TRIM65)

Tumor metastasis involves the reorganization of the cytoskeleton, whose activities are controlled by GTPases^31^. When bound to guanosine diphosphate (GDP), GTPases are inactivated, which is regulated by GTPase-activating protein (GAP)^32^. Rho A belongs to the Rho family of GTPases and regulates the cytoskeleton. Rho GTPase-activating protein 35 (ARHGAP35), a Rho GAP, regulates polarized cell migration and inhibits Rho GTPase. In colorectal cancer (CRC), E3 Ub ligase TRIM65 ubiquitinates and degrades ARHGAP35, which leads to subsequent elevated Rho GTPase activity and cytoskeleton remodeling 33 (Fig. 2). The TRIM65-ARHGAP35-Rho A axis enhances cancer cell migration by modulating the actin cytoskeleton.

### F-box only protein 22 (FBXO22)

FBXO22, one of the F-box-only proteins, is the substrate-recognizing subunit of the SCF E3 Ub ligase complex 34. FBXO22 ubiquitinates nuclear PTEN at lysine 221 (K221). Nuclear PTEN exerts potent tumor inhibition capacity. In CRC tissues, FBXO22 overexpression contributes to the downregulation of nuclear PTEN to promote tumorigenesis, which is reversed by the mutation of K221 35. FBXO22 is upregulated and negatively correlated with p21 in HCC. FBXO22 functions as an oncogene by mediating the ubiquitination and degradation of p21 to promote HCC pathogenesis and progression 36. FBXO22 mediates Lys-63-linked liver kinase B1 (LKB1) polyubiquitination and inhibits LKB1-AMPK-mTOR signaling in lung adenocarcinoma 37. FBXO22 promotes melanoma angiogenesis and migration of tumor cells via upregulating HIF-1α and vascular endothelial growth factor A (VEGFA) 38 (Fig. 2).

## DUBs

DUBs can reverse ubiquitination by cleaving the isopeptide bond between the Ub and the substrate. Currently, over 100 DUBs have been identified and can be divided into six subclasses: i) Ub-specific proteases (USPs); ii) ovarian tumor proteases (OTUs); iii) Ub C-terminal hydrolases (UCHs); iv) Machado-Joseph disease proteases; v) JAB1/MPN/Mov34 metalloenzymes; and vi) monocyte chemotactic protein-induced protein^39^. DUBs act as tumor suppressors or oncogenes and play essential roles in regulating various types of tumors (Table 1). DUBs have emerged as promising therapeutic targets in cancer.

### DUBs inhibiting metastasis

#### Ub carboxyl-terminal hydrolase BAP1 (BAP1)

**Figure 3:**
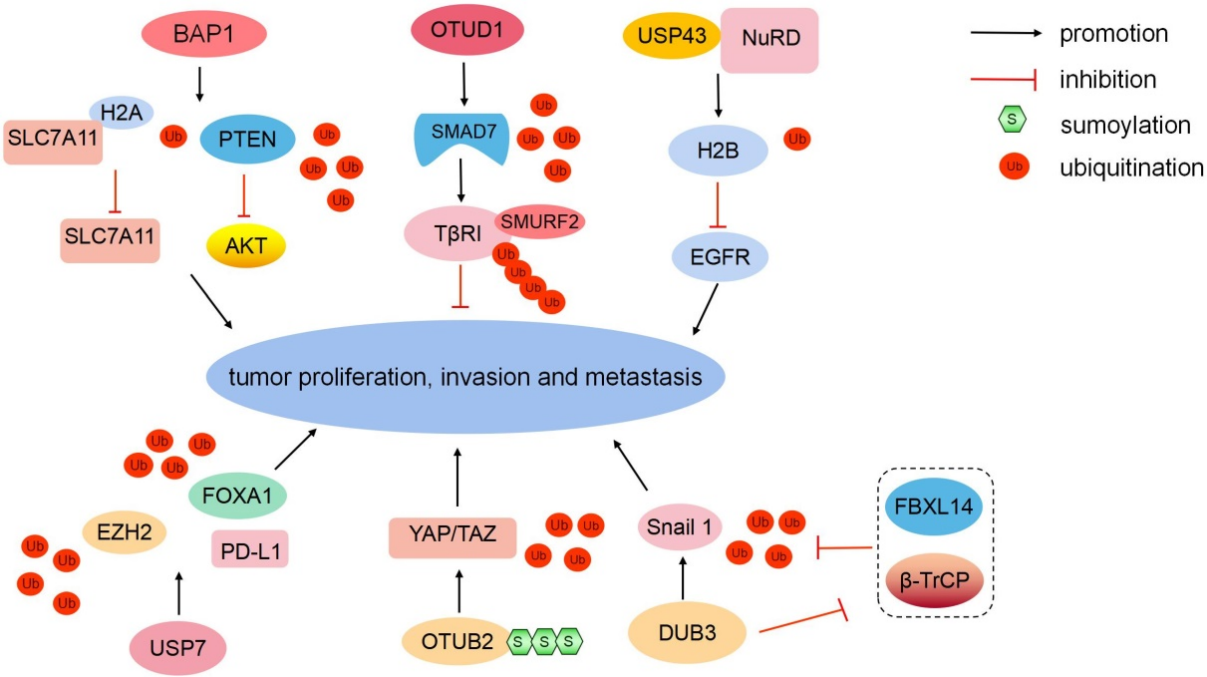
** Different DUBs regulate tumorigenesis.** BAP1 can deubiquitinate PTEN and reduce H2AUb on the SLC7A11 promoter to inhibit tumor development and metastasis. OTUD1 can eliminate the K48-linked Ub chain from SMAD7. The USP43-NuRD complex catalyzes H2BK120 deubiquitination to suppress downstream EGFR. USP7 can stabilize EZH2 and FOXA1 via deubiquitination to promote tumor growth. USP7 upregulates PD-L1 expression in the tumor microenvironment. SUMOylated-OTUB2 deubiquitinates YAP/TAZ to potentiate tumor cell proliferation and metastasis in a Hippo-independent manner. Dub3 can stabilize Snail1 via deubiquitination and inhibiting β-TrCP1 and FBXL14-mediated ubiquitination degradation.

BAP1 belongs to the UCH domain-containing proteins, and it can physically bind to and deubiquitinate PTEN to stabilize PTEN protein. Downregulated BAP1 leads to the decrease of PTEN protein levels and the activation of the Akt signaling pathway, therefore promoting malignant transformation and metastasis in prostate cancer. Clinically, low BAP1 expression is positively correlated with aggressive prostate tumor proliferation and lymphatic metastasis 40. In ICC, BAP1 inhibits ERK1/2 and JNK/c-Jun pathways 41. Moreover, BAP1 mediates the metabolic regulation of ferroptosis and tumor suppression. BAP1 reduces histone 2A ubiquitination (H2AUb) on the cystine/glutamate transporter (SLC7A11) promoter and represses SLC7A11 expression in a deubiquitination-dependent manner 42. The loss of cystine transport mediated by SLC7A11 induces ferroptosis to inhibit tumor development and metastasis 43 (Fig. 3). In summary, as a major tumor suppressor, mutated BAP1 is associated with numerous human malignancies, which is defined as “BAP1 cancer syndrome” 44.

#### OTU domain-containing protein 1 (OTUD1)

In the TGF-β signaling pathway, SMAD7 recruits E3 ligase SMURF2 to TGF-β type I receptor (TβRI) 45. However, E3 Ub-protein ligases RNF12 and Itchy homolog can degrade SMAD7 to antagonize its suppression 46, 47. In breast cancer, OTUD1 can selectively eliminate the K48-linked Ub chain from SMAD7 to stabilize it (Fig. 3). Moreover, OTUD1 can cleave the K33-linked Ub chain on the lysine 220 site and unveil the PY motif of SMAD7 48. The exposed SMAD7 PY motif subsequently binds the WW domain of SMURF2 to degrade TβRI via ubiquitination 45, 49. In summary, OTUD1 suppresses the TGF-β-induced metastasis by exerting dual effects on SMAD7.

#### Ub carboxyl-terminal hydrolase 43 (USP43)

The EGFR/PI3K/AKT pathway is aberrantly activated in various cancers 50. PTEN can negatively regulate AKT kinase activity 51. In addition, another molecular regulatory mechanism exists. Nuclear USP43 is physically associated with the nucleosome remodeling and deacetylase (NuRD) complex. The USP43-NuRD complex coordinately catalyzes H2BK120 deubiquitination to suppress downstream EGFR 52 (Fig. 3). Simultaneously, activated AKT can phosphorylate cytoplasmic USP43 on Ser29, phosphorylated USP43 is detained in the cytoplasm, reduced nuclear USP43 and accumulated EGFR potentiate the EGFR/PI3K/AKT pathway to promote breast cell proliferation and invasion 52. USP43 is a hub of the USP43-NuRD complex, the reciprocally inhibitory loop between the USP43-NuRD complex and the EGFR/PI3K/AKT pathway synergistically modulates breast carcinogenesis. The ratio of nuclear/cytoplasmic USP43 is a worthy prognostic indicator 52.

### DUBs promoting metastasis

#### USP7

USP7 can coordinate with histone-lysine N-methyltransferase EZH2 (EZH2)-catalyzed methylation to remove ubiquitination and enhance FOXA1 protein stability, promoting prostate cancer growth 53. Moreover, USP7 can stabilize EZH2 via deubiquitination 54 (Fig. 3). USP7 is upregulated in M2 macrophages. USP7 inhibition can induce the polarization of tumor-associated macrophages from M2 into M1 by activating the P38 MAPK pathway and upregulating the expression of programmed cell death 1 ligand 1 (PD-L1) in the tumor microenvironment 55. Therefore, USP7 blockade combined with anti-PD-1 immunotherapy exert an inhibitory effect on tumors in lung cancer.

#### SUMOylated-Ub thioesterase OTUB2 (OTUB2)

Transcriptional coactivator YAP1 (YAP) and tafazzin (TAZ) are generally downregulated by the canonical Hippo pathway 56. However, YAP and TAZ are hyperactivated and induce tumor proliferation and metastasis, while the Hippo pathway is still active in multiple malignancies, including breast cancer 57, 58. DUB OTUB2 mediates the activation of YAP and TAZ in a Hippo-independent manner (Fig. 3). Mechanistically, OTUB2 is poly-SUMOylated at lysine 233 (K233), SUMOylated-OTUB2 can subsequently bind YAP/TAZ through SUMO-interacting motif in YAP and TAZ 58. OTUB2 deubiquitinates and activates YAP and TAZ, and accumulated YAP and TAZ translocate into the nucleus in which they interact with TEA domain family transcription factors and transcriptionally activate genes to potentiate cell proliferation and metastasis 59, 60. Meanwhile, activated EGF-RAS signaling strengthens OTUB2 SUMOylation and elevates YAP/TAZ protein levels to promote cancer stemness and metastasis 58, 61. In summary, the novel SUMOylated-OTUB2-mediated regulatory mechanism expands the complexity of YAP/TAZ beyond the Hippo pathway. OTUB2 may be a potential drug target to suppress cancer progression for patients harboring RAS mutations.

#### Dub3

Snail1 is a critical EMT-driving transcription factor and confers tumor metastatic and cancer stem cell-like properties 62. The E3 ligases β-TrCP1 and F-box/LRR-repeat protein 14 (FBXL14) can degrade Snail1 via ubiquitination 63. In breast cancer, Dub3 accounts for Snail1 stabilization, and inflammatory cytokine IL-6 can increase the expression of Dub3. Meanwhile, Dub3 also inhibits the activity of β-TrCP1 and FBXL14 to block Snail1 ubiquitination 64 (Fig. 3). Overall, Dub3 senses inflammatory stimulation and converts it into Snail1 stabilization.

## Conclusion

Although this brief review only scratches the surface of ubiquitination and deubiquitination in cancer, it highlights the significance of E3s and DUBs in a range of processes involved in tumor progression. Ubiquitination components are potential therapeutic targets for cancer treatment 65. However, several issues remain obstacles for targeted therapy. DUBs share similar structural characteristics among family members, and ubiquitination involves substantial conformational changes. We still endeavor to deal with the challenges ahead, such as defining novel E3s, DUBs, and targeted substrates, investigating whether there exists unknown crosstalk among distinct E3s or DUBs, and decoding the unknown pathways linking ubiquitination with other cellular physiological mechanisms. Several valuable E3s or DUBs are promising clinical prognostic indexes and drug targets. Proteolysis Targeting Chimeras (PROTACs) exploit the intracellular Ub-proteasome system to degrade target proteins 66, selectively. In tumor xenografts, small-molecule PROTACs can significantly attenuate tumor progression 67.

This review provides a glimpse into the importance and extensiveness of ubiquitination component-mediated tumor invasion and metastasis, which represents a worthy research prospect.

## Figures and Tables

**Table 1:** A brief overview of different DUBs in various cancers

DUBs	Biological effect	Brief biological mechanism	Involvement in cancer	Refs
USP1	Oncogene	Phosphorylated USP1 (via ATM/ATR) deubiquitinates and stabilizes Snail.	USP1 induces platinum resistance, cancer cell stemness, and metastatic dissemination in ovarian cancer.	68
		USP1 deubiquitinates KPNA2 and enhances pro-metastatic genes expression.	The intervention of USP1 via pimozide or ML323 suppresses metastasis.	69
		USP1 deubiquitinates and increases TAZ protein stability.	Loss of USP1 reduces TAZ to inhibit cell proliferation and migration, and USP1 is a potential therapeutic target in triple-negative breast cancer (TNBC).	70
		USP1 deubiquitinates and stabilizes ribosomal protein S16 (RPS16).	USP1-mediated RPS16 stabilization promotes cell proliferation and metastasis in hepatocellular carcinoma (HCC).	71
USP2a	Oncogene	On TGF-β stimulation, USP2a deubiquitinates TGFBR1 (K33-linked ubiquitin chain), recruiting SMAD2. TGFBR2 subsequently phosphorylates USP2a, facilitating SMAD2 into the cytoplasm.	It is associated with trans-activating EMT genes to promote metastasis in lung adenocarcinomas.	72
		USP2a deubiquitinates and stabilizes RAB1A.	USP2a is highly upregulated and promotes hepatocellular carcinoma (HCC) cell progression.	73
USP3	Oncogene	USP3 deubiquitinates and upregulates SUZ12 protein expression.	USP3 promotes TGF-β1-induced EMT and cell migration in gastric cancer.	74
USP4	Oncogene	USP4 deubiquitinates and stabilizes Twist1 protein.	USP4 enhances cancer cell stemness which mediates tumor development and metastasis in lung cancer and breast cancer.	75
		USP4 constitutes the PAK5-DNPEP-USP4 axis. Aberrant PAK5 phosphorylates DNPEP, which preferentially facilitates DNPEP ubiquitination degradation, in turn inhibiting DNPEP-mediated USP4 downregulation.	USP4 elicits cancer cell proliferation, invasion, and metastasis in breast cancer.	76
USP5	Oncogene	USP5 deubiquitinates and stabilizes SLUG.	USP5 promotes EMT, tumor growth, and metastasis, rescuing by Formononetin targeting USP5 in hepatocellular carcinoma (HCC).	77
		USP5 prohibits β-catenin ubiquitination degradation and upregulates β-catenin, which activates the Wnt/β-catenin signaling.	USP5 is overexpressed in non-small cell lung cancer to promote EMT, invasion, and metastasis.	78
USP6	Oncogene	USP6 promotes invasion and metastasis, and acts as an efficient prognostic biomarker.	USP6 is highly overexpressed in colon cancer.	79
USP7 (HAUSP)	Oncogene	USP7 promotes the circulation of tumor cells (CTCs) to reside in the bone marrow.	Inhibition of USP7 can arrest bone marrow-resident tumor cells (BMRTC) in BM and decrease metastasis. USP7 could be a therapeutic target in melanoma.	80
		K63-polyubiquitinated HAUSP deubiquitinates and stabilizes HIF-1α, and causes CBP-mediated H3K56 acetylation to regulate HIF-1α target gene promoters.	Under hypoxia, E3 ligase HectH9 is required for K63-polyubiquitinated HAUSP to promote EMT and metastasis in lung cancer.	81
		USP7 promotes proliferation and invasion.	Overexpressed USP7 represents a worse overall survival and acts as an independent prognostic indicator in epithelial ovarian cancer (EOC) and oral squamous cell carcinoma (OSCC).	82, 83
		USP7 overexpression activates the PI3K/AKT signaling pathway.	The USP7 inhibitor P5091 suppresses cell proliferation and metastasis and promotes hepatoblastoma (HB) apoptosis.	84
		USP7 activates the Wnt/β-catenin signaling pathway.	USP7 inhibitor P22077 induces apoptosis and DNA damage and suppresses cell migration and invasion in melanoma.	85
USP8	Oncogene	Overexpressed USP8 increases p-AKT, activates AKT signaling, and regulates intrinsic apoptosis pathway.	USP8 suppresses apoptosis and promotes proliferation, invasion, and metastasis in cholangiocarcinoma.	86
	TSG (Tumor suppressor gene)	USP8 is a protective factor and prognosticates better clinical outcomes.	USP8 is downregulated in breast cancer.	87
USP9X	Oncogene	USP9X activates EMT.	USP9X overexpression promotes invasion and migration, and inhibits apoptosis in pancreatic ductal adenocarcinoma (PDAC).	88,89
		USP9X bans TIF1γ from ubiquitinating SMAD4 and maintains its nuclear retention to induce the TGF-β signaling.	Plasma-free fatty acids (FFA) promote the SMAD4-USP9X interaction via ERK to elicit TGF-β-induced metastasis for obese breast cancer patients.	
USP10	Oncogene	USP10 deubiquitinates Smad4 (K48) and activates TGF-β.	USP10-siRNA and Spautin1 inhibitor can downregulate USP10 to suppress Smad4 and metastasis in hepatocellular carcinoma.	90
		USP10 deubiquitinates NLRP7 to induce M2 TAM polarization via CCL2 secretion.	USP10 is highly expressed and stabilizes NLRP7 to promote cell proliferation and metastasis in colorectal cancer.	91
USP11	Oncogene	USP11 deubiquitinates and stablizes PPP1CA to activate the ERK/MAPK pathway.	USP11 is overexpressed and promotes metastasis in colorectal cancer.	92-95
		USP11 deubiquitinates and stabilizes nuclear factor (NF90).	USP11 can promote proliferation and metastasis in hepatocellular carcinoma.	
		USP11 stabilizes TGFβ receptor type 2 (TGFBR2).	USP11 enhances TGFβ-induced EMT to promote breast cancer metastasis.	
		USP11 is an independent prognostic predictor.	USP11 is overexpressed and promotes migration and metastasis in hepatocellular carcinoma.	
	TSG	USP11 deubiquitinates ARID1A and prevents its degradation to inhibit SDC2 activation.	USP11 antagonizes with TRIM32 to stabilize ARID1A and to suppress proliferation and metastasis in squamous cell carcinomas (SCCs).	96, 97
		USP11 deubiquitinates and stabilizes PTEN and subsequently suppresses the PI3K/AKT pathway.	PTEN inhibits the PI3K/AKT-mediated phosphorylation of FOXO to increase its nuclear localization and to enhance USP11 transcription. The PTEN-PI3K/AKT-FOXO-USP11 regulatory feedforward loop regulates the tumor- suppressive activity of PTEN.	
USP12	Oncogene	USP12 deubiquitinates and stabilizes midkine (MDK).	The USP12-MDK axis promotes angiogenesis to faciliate breast cancer metastasis.	98
USP14	Oncogene	USP14 deubiquitinates PI3K.	USP14 inhibitor Lidocaine (Lido) suppresses proliferation and migration while aggravating hepatocellular carcinoma cell apoptosis.	99
		USP14 overexpression promotes proliferation and migration and prevents apoptosis.	USP14 is remarkably upregulated in pancreatic ductal adenocarcinoma (PDAC).	100
USP15	Oncogene	USP15 overexpression promotes proliferation and prevents apoptosis.	High USP15 expression indicates a worse prognosis, and USP15 could be a therapeutic target in hepatocellular carcinoma.	101
		USP15 promotes β-catenin nuclear translocation and activates the Wnt/β-catenin pathway.	USP15 is upregulated and promotes EMT, cell proliferation, and metastasis.	102
USP18	Oncogene	USP18 deubiquitinates ZEB1.	USP18 is overexpressed and induces ZEB1-mediated EMT to promote metastasis in esophageal squamous cell carcinomas (ESCC).	103
USP20	Oncogene	USP20 deubiquitinates β-catenin.	USP20 highly expresses and regulates the Wnt/β-catenin pathway to potentiate tumorigenesis in colon cancer.	104
USP21	Oncogene	USP21 deubiquitinates EZH2.	USP21 upregulates and promotes EMT and metastasis in bladder cancer.	105, 106
		USP21 deubiquitinates Fos-related-antigen-1 (Fra-1) and enhances AP-1 target gene expression.	USP21 overexpresses and promotes Fra1-dependent metastasis in colorectal cancer.	
USP22	Oncogene	USP22 activates AP4 transcription to induce EMT.	USP22 and AP4 overexpress and promote liver metastasis in colorectal cancer.	107-111
		USP22 stabilizes BMI1 protein to maintain cancer stemness.	USP22 and BMI1 overexpress and facilitate proliferation in gastric cancer.	
		High USP22 enhances angiogenesis, metastasis, and recurrence.	USP22 knockout suppresses metastasis and sensitizes cisplatin and irradiation in non-small cell lung cancer.	
		USP22 increases the relative abundance of myeloid cells vs. cytotoxic T cells via its deubiquitinase activity.	USP22 ablation can suppress metastasis and improve the response to immunotherapy in pancreatic ductal adenocarcinoma (PDA).	
		USP22 promotes gastric cancer progression by modulating FOXO1 and the YAP signaling pathways via c-Myc/NAMPT/SIRT1.	USP22 is overexpressed, and its depletion suppresses invasion and metastasis in gastric cancer.	
	TSG	USP22 decreases mTOR activity.	USP22 deficiency activates mTOR and tumorigenesis, reversed by mTOR inhibitor treatment in colorectal cancer.	112
USP25	Oncogene	mi-RNA 200c reduces the USP25 gene mRNA and protein levels to inhibit invasion and migration.	USP25 protein and mRNA levels are highly expressed in non-small cell lung cancer.	113
USP26	Oncogene	USP26 deubiquitinates and stabilizes Snail.	USP26 is highly expressed in esophageal squamous cell carcinoma (ESCC).	114
USP28	Oncogene	USP28 stabilizes lysine specific demethylase1.	USP28 is overexpressed in gastric cancer.	115, 116
		USP28 antagonizes GSK3β-Fbw7-dependent HIF-1α ubiquitination degradation to affect HIF-1α-dependent angiogenesis and carcinogenesis.	Expression of USP28 is elevated in colon and breast carcinomas.	
USP29	Oncogene	USP29 interacts simultaneously with Snail and SCP1 to stabilize Snail via deubiquitination and dephosphorylation.	TNFα, TGFβ, and Hypoxia can induce USP29 to promote gastric cancer cell migration.	117
USP33	Oncogene	USP33 deubiquitinates specificity protein 1 (SP1) to upregulate c-met.	USP33 is overexpressed and is a prognostic biomarker and therapeutic target in hepatocellular carcinoma.	118, 119
	TSG	USP33 can deubiquitinate and stabilize Robo1 to inhibit EMT and cell migration in a Slit-Robo pathway-dependent manner.	USP33 expression is downregulated and it is an independent prognostic marker in colorectal cancer and gastric cancer.	120, 121
USP37	Oncogene	USP37 deubiquitinates Snail.	Upregulated expression of USP37 promotes lung cancer cell migration.	122-124
		USP37 stabilizes the hedgehog (Hh) pathway component Gli-1.	USP37 can regulate the stemness, cell invasion, cisplatin sensitivity, and EMT via the Hh pathway in breast cancer.	
		USP37 binds and deubiquitinates Snai1.	Overexpression of USP37 upregulates Snai1 to promote cancer cell migration.	
USP43	TSG	USP43 physically binds to the chromatin remodeling NuRD complex and catalyzes H2BK120 deubiquitination to repress the EGFR gene.	EGFR/PI3K/Akt-mediated phosphorylated USP43 binds to the 14-3-3β/ε heterodimer and sequestrates in the cytoplasm to drive breast carcinogenesis.	52
USP44	Oncogene	USP44 deubiquitinates EZH2, a histone H3 lysine 27 methyltransferase.	USP44 knockdown decreases the EZH2 protein level and inhibits prostate cancer cells' tumorigenesis and cancer stem cell-like behaviors.	125, 126
		USP44 expression in breast cancer stem cells (CSC) contributes to the formation of vasculogenic mimicry (VM) to promote transendothelial migration.	USP44 silencing abates VM and USP44^+^CSC subclones act as an independent prognostic biomarker in breast cancer.	
USP46	Oncogene	USP46 deubiquitinates ENO1 and promotes EMT.	USP46 is overexpressed in esophageal squamous cell carcinoma (ESCC).	127
USP47	Oncogene	USP47 deubiquitinates and stabilizes Snail to induce EMT.	Inhibiton of USP47 with P5091 can reverse the EMT phenotype.	128-130
		USP47, as a novel target of Sox9, mediates hypoxia-induced EMT via deubiquitinating Snail.	The expression of USP47 is elevated, and silencing USP47 can promote Snail degradation and attenuate EMT in colorectal cancer.	
		USP47 abrogates the SMURF2-mediated ubiquitination of special AT-rich sequence-binding protein-1 (SATB1) to promote colon cacer cell proliferation and metastasis.	USP47 depletion sensitizes colon cancer cells to 5-FU treatment-induced apoptosis.	
USP48	Oncogene	USP48 promotes migration and invasion.	Ablation of USP48 increases the responsiveness to carboplatin treatment in ovarian cancer.	131
USP51	Oncogene	CDK4/6-mediated phosphorylated USP51 can deubiquitinate and stabilize ZEB1 to induce EMT.	The overexpressed p-USP51 is correlated to a poor prognosis for breast cancer patients, and the CDK4/6-USP51-ZEB1 axis could be a viable therapeutic target.	132-134
		USP51 increases FAT4 protein level and is imperative for FAT4's function.	USP51 suppression contributes to the inhibition of FAT4 and promotes proliferation and invasion of endometrial cancer (EC).	
USP54	Oncogene	USP54 is of pro-tumorigenic properties.	USP54 is upregulated in colorectal carcinoma and is a promising therapeutic target.	135
OTUB1	Oncogene	OTUB1 stabilizes Snail to promote metastasis.	OTUB1 is highly expressed in esophageal squamous cell carcinoma (ESCC), and higher expression of OTUB1 predicts poor prognosis.	136, 137
		OTUB1 induces EMT to promote metastasis.	OTUB1 is overexpressed and related to poor survival and serves as an independent prognostic factor in colorectal cancer (CRC).	
OTUB2	Oncogene	OTUB2 can deubquitinate U2AF2 and activate the AKT/mTOR pathway.	OTUB2 and U2AF2 are highly expressed and associated with metastasis and poor survival. OTUB2 may serve as a potential prognostic indicator and therapeutic target in NSCLC.	138, 139
		EGF/KRAS-induced SUMOylation of OTUB2 can deubiquitinate and activate YAP/TAZ.	OTUB2 can promote cancer stemness and metastasis via the Hippo-independent pathway.	
OTUD1	TSG	OTUD1 deubiquitinates K48-linked and K33-linked SMAD7 to enhance SMURF2 binding to suppress TGFβ.	High-level OTUD1 inhibits TGFβ-induced cancer stemness and metastasis in breast cancer.	48
OTUD3	Oncogene	OTUD3 stabilizes GRP78 to promote lung tumorigenesis, reversed by CHIP which can ubiquitinate OTUD3.	CHIP knockdown increases lung cancer cell invasion in an OTUD3 and GRP78- dependent manner.	140
	TSG	OTUD3 deubiquitinates and stabilizes PTEN.	Reduction of OTUD3 causes decreased PTEN abundance and correlates with breast cancer progression.	141
OUTD6B	TSG	OTUD6B couples pVHL to form the CBC^VHL^ complex to decrease its ubiquitination degradation, thereby attenuating HIF-1α.	OTUD6B is positively correlated with pVHL, but negatively with HIF-1α and vascular endothelial growth factor in hepatocellular carcinoma.	142
OUTD7B	TSG	OTUD7B promotes proliferation and metastasis via the Akt/VEGF signal pathway.	OTUD7B is highly expressed in lung squamous carcinoma and adenocarcinoma, and correlates with a worse prognosis, and may be an independent predictive indicator.	143
BAP1	Oncogene	BAP1 deubiquitinates transcription factor KLF5.	BAP1 konckdown inhibits breast cancer tumorigenicity and lung metastasis, and BAP1 could be a therapeutic target.	144
	TSG	BAP1 inhibits the ERK1/2 and JNK/c-Jun pathway to repress intrahepatic cholangiocarcinoma (ICC).	mRNA and protein level of BAP1 are downregulated, related to ICC aggressive characteristics. BAP1 may be a prognostic and therapeutic target.	41, 145-147
		BAP1 mutants induce migration.	BAP1 blocks metastasis in solid pseudopapillary neoplasms (SPN).	
			BAP1 somatic mutations, not germline mutations, infrequently occur in uveal melanoma.	
CYLD	TSG	CYLD regulates genes involved in proliferation, migration, and angiogenesis.	CYLD-deficiency enhances melanoma progression.	148-150
		CYLD can reverse the K63 ubiquitination of c-Jun and c-Fos to repress the JNK/AP1 pathway.	CYLD mutant enhances squamous cell carcinoma growth and migration in an AP1-dependent manner.	
		Snail1 inhibits CYLD to promote BCL-3 nuclear translocation, activating cycling D1 and N-cadherin.	Upregulation of CYLD expression can repress proliferation and invasion in melanoma.	
UCHL1	Oncogene	UCHL1 promotes EMT.	UCHL1 is overexpressed, and knockdown can induce MET in metastatic prostate cancer.	151-156
		UCHL1 activates the AKT/MAPK pathway.	AKT negative mutant and silencing UCHL1 suppress invasion and metastasis in non-small cell lung cancer.	
		UCHL1 activates the MAPK/ERK pathway.	ERK inhibitor U0126 can block multidrug resistance and invasion in UCHL1-overexpressed breast cancer cells.	
		UCHL1 compromises VHL-mediated ubiquitination of HIF-1α to promote metastasis.	UCHL1 is overexpressed in breast and lung cancer. It may be a prognostic marker and therapeutic target.	
		UCHL1 expression is positively associated with renal cell cancer's (RCC) metastatic phenotype.	UCHL1 might serve as a potential diagnostic and prognosis biomarker for RCC patients.	
		UCHL1 deubiquitinates TGFβ type I receptor and SMAD2.	The UCHL1 inhibitor 6RK73 suppresses TGFβ-induced metastasis, and UCHL1 could potentially target triple-negative breast cancer (TNBC) treatment.	
UCHL5	Oncogene	UCHL5 can activate the Wnt/β-catenin pathway and upregulate β-catenin.	UCHL5 is overexpressed, and promotes tumorigenesis and growth in endometrial cancer (EC), which can be abrogated by the Wnt/β-catenin pathway inhibitor XAV939.	157
COPS5	Oncogene	COPS5 deubiquitinates HK2 and attenuates its degradation to regulate glycolysis.	COPS5 relates to HK2 overexpression, and Curcumin can inhibit CSN5 activity to decrease HK2, and to repress glycolysis and metastasis in hepatocellular carcinoma.	158, 159
		COPS5 deubiquitinates and stabilizes ZEB1.	COPS5 expression is elevated and its knockdown can suppress EMT and metastasis.	
COPS6	Oncogene	COPS6 increases CHIP self-ubiquitination to elevate EGFR stability.	COPS6 is overexpressed and the CSN6-CHIP-EGFR axis could be a therapeutic target in glioblastoma.	160, 161
		COPS6 inhibits the autophagy of CathepsinL (CTSL) via the mTOR pathway.	COPS6 and CTSL are overexpressed and indicate aggressive cervical cancer.	
ATXN3	Oncogene	ATXN3 deubiquitinates KLF4.	High ATXN3 and KLF4 expression are associated with a poor prognosis in breast cancer patients.	162

## Abbreviations

Ub: ubiquitin
DUBs: deubiquitinases
SKP2: S phase kinase-associated protein 2
FBXW2: F-box and WD-repeat domain-containing 2
TAK1: transforming growth factor-β-activated kinase 1
β-TrCP1: β-transducin repeat-containing protein 1
FBW7: F-box and WD repeat domain-containing 7
Brg1: transcription activator Brg1
Mcl-1: induced myeloid leukemia cell differentiation protein Mcl-1
YTHDF2: YTH domain-containing family protein 2
EMT: epithelial-mesenchymal transition
CK1: casein kinase 1
UBR7: putative E3 Ub-protein ligase UBR7
CDH4: cadherin-4
HIF-1α: hypoxia-inducible factor 1α
PHGDH: phosphoglycerate dehydrogenase
PKM: pyruvate kinase PKM
UBE3C: Ub-protein ligase E3C
AHNAK: neuroblast differentiation-associated protein AHNAK
TRIM65: tripartite motif-containing protein 65
ARHGAP35: Rho GTPase-activating protein 35
FBXO22: F-box only protein 22
LKB1: liver kinase B1
VEGFA: vascular endothelial growth factor A
BAP1: Ub carboxyl-terminal hydrolase BAP1
H2AUb: histone 2A ubiquitination
SLC7A11: cystine/glutamate transporter
SMURF2: E3 Ub-protein ligase SMURF2
TβRI: TGF-β type I receptor
OTUD1: OTU domain-containing protein 1
USP43: Ub carboxyl-terminal hydrolase 43
NuRD: nucleosome remodeling and deacetylase
H2BK120: histone H2B at lysine 120
USP: Ub-specific protease
EZH2: histone-lysine N-methyltransferase EZH2
PD-L1: programmed cell death 1 ligand 1
OTUB2: Ub thioesterase OTUB2
YAP: transcriptional coactivator YAP1
TAZ: tafazzin
FBXL14: F-box/LRR-repeat protein 14
